# Multi-Frequency Intraoperative Monitoring of Hearing Preservation during Cochlear Implantation

**DOI:** 10.3390/life12050636

**Published:** 2022-04-25

**Authors:** Piotr Henryk Skarżyński, Artur Lorens, Adam Walkowiak, Marek Polak, Henryk Skarżyński

**Affiliations:** 1 World Hearing Center, Institute of Physiology and Pathology of Hearing, 02-042 Warsaw, Poland; a.lorens@ifps.org.pl (A.L.); a.walkowiak@ifps.org.pl (A.W.); h.skarzynski@ifps.org.pl (H.S.); 2 Institute of Sensory Organs, 05-830 Kajetany, Poland; 3 Center of Hearing and Speech Medincus, 05-830 Kajetany, Poland; 4 Heart Failure and Cardiac Rehabilitation Department, Second Faculty of Medicine, Medical University of Warsaw, 03-242 Warsaw, Poland; 5 R&D Med-El, Furstenweg 77A, 6020 Innsbruck, Austria; marek.polak@medel.com

**Keywords:** electrocochleography, cochlear implant, partial deafness treatment (PDT)

## Abstract

(1) Background: Current indications for cochlear implants (CIs) have expanded to include patients with appreciable low-frequency hearing. However, longitudinal results indicate that only one-third of these recipients retain full hearing preservation. In another words, the remaining two-thirds lose this facility either partially or fully. This points to the need to better understand the impact of cochlear implantation on cochlear integrity. Intracochlear electrocochleography (ECochG) involves the recording of electrical potentials generated in the inner ear in response to acoustic stimuli, and previous studies have shown that these potentials give an indication of residual inner ear function. Aim of the research: The aim is to monitor intracochlear ECochG during CI surgery and gain a better understanding of how the implant impacted inner ear function. A newly developed SPL Chirp was used for stimulation. (2) Methods: Intracochlear ECochG signals were measured in a subject with residual preoperative low-frequency hearing, while an electrode array was introduced into the cochlea and was continued until the round window was sealed. Afterwards, surgical events were reviewed with the surgeon; preoperative and postoperative radiological data and hearing thresholds were also evaluated. (3) Conclusions: Real-time intraoperative monitoring, with multifrequency evaluation and video recording, has the potential to allow surgeons and audiologists to continuously assess cochlear function. ECochG monitoring may be a useful tool during cochlear implantation to gain frequency-specific information on the status of the patient’s hearing, assisting surgeons to lower hearing trauma during the operation.

## 1. Introduction

Electrocochleography (ECochG) measures the electrical potentials generated by the inner ear in response to acoustic stimulation. It is therefore a reflection of the remaining inner ear function [1]. In a cochlear implant (CI) patient, a recording can be made directly through the cochlear implant electrode, which is connected to a telemetry system [2,3]. This allows data collection during electrode insertion, and near to real-time measurement, immediately post-insertion, as well as at subsequent clinical follow-ups. 

The insertion of an electrode into the cochlea usually causes a certain degree of trauma to the delicate inner ear structure (electrode insertion trauma, EIT). Inner ear injury during electrode insertion can be caused by: (1) direct mechanical trauma from the electrode or hydraulic forces; (2) fracturing of the osseous spiral lamina; (3) injury to the modiolus; (4) compression or tearing of superficial arteries or veins; (5) damage to the lateral wall; (6) misdirection of the electrode into the scala media or scala vestibuli; (7) disruption of labyrinthine fluid homeostasis through excessive suctioning of perilymph; (8) introduction of blood into the scala tympani [4].

It is generally assumed that the amount of EIT correlates with the level of postoperative hearing preservation, so that the extent of hearing preservation serves as a good indicator of cochlear integrity. Previous work has demonstrated that ECochG recordings can be correlated to postoperative hearing measures [5,6,7]. However, all previous studies have used a single frequency for stimulation. The current study uses a novel chirp signal developed from in vivo measurements in humans [8], which allowed us to acquire multi-frequency information during insertion. In this way, we can continuously monitor the intracochlear ECochG during electrode insertion, packing of the round window and electrode maneuvering. In this paper, the electrographic findings are analyzed, in conjunction with the surgical video, in order to give some insights into the potential causes of EIT. 

## 2. Materials and Methods

### Case Report

The subject, 47 years old, received a Sonata 2 cochlear implant (Med-El Corporation, Innsbruck, Austria) employing a Flex 28 electrode array. This array has 12 electrodes, with 2.1 mm between each one, for a total length of 28 mm. The surgical approach accorded with the 6-step surgical technique [9,10], in which the array is inserted through the round window and the aim is to preserve middle ear structures [9,10]. In certain cases, the endomieteal approach could be considered for having a better access to the round window [11]. The electrode array was inserted fully into scala tympani via the round window on the right side. The patient provided informed, written consent for their participation in the study and for the publication of its findings.

To evaluate the trauma caused by insertion of electrodes into scala tympani, we assessed the difference between preoperative and postoperative audiograms. The preoperative audiogram was obtained 1–4 weeks prior to cochlear implantation. The postoperative audiogram was obtained at the time of the initial activation of the audio processor.

**Figure 1:**
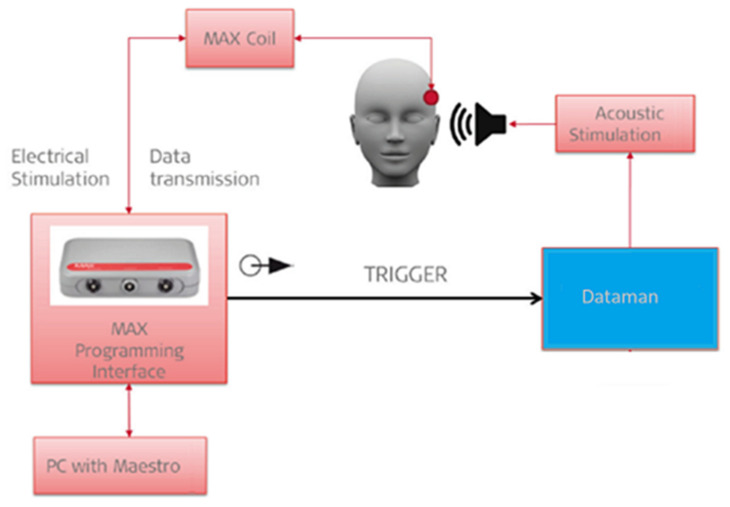
Setup of the intracochlear ECochG measurements. Acoustic simulation is delivered by an insert ear phone; inner ear responses are picked up by the implant electrode and passed to a telemetry and recording system.

Intracochlear ECochG measurements were performed with the Med-El EAEP measurement tool (see Figure 1). Stimulation was delivered by E-A-RLINK™ insert earphones (3M, St. Paul, MN, USA). To provide acoustic stimulation, a Dataman system (Dataman Programmers Ltd., Dorset, UK) was used. The ECoshG recordings were performed with the implant electrode and back-telemetry EAEP measurement system.

Preoperative computed tomographic imaging (CT) was performed 1 month before the surgical intervention. CT images were evaluated using Otoplan v 3.1 software (CA Scination AG, Bern, Switzerland), from which cochlear parameters were measured and the required location of the intracochlear electrodes estimated. Based on this evaluation, the electrode length was selected. Since the patient had substantial preoperative hearing, an electrode length of 28 mm was selected, corresponding to a calculated full electrode insertion angle of 490°. Another reason for deciding on the Flex 28 electrode was that the subject had already been implanted on the left side with this array. These data were imported into the Maestro 9 AS software. After an uneventful surgery, a postoperative CT was obtained, from which the location of the intracochlear electrode contacts and associated cochlear parameters were measured. 

For stimulation, a novel SPL Chirp signal, developed from in vivo measurements in humans [8], was used. The duration of the SPL Chirp is 12 ms, and contains frequencies of 0.25, 0.5, 1, 2 and 4 kHz. For easy comparison with the audiogram, the signal was calibrated in HL levels for every frequency component of the SPL Chirp signal. In order to avoid spectral splatter, a Blackman window with rise and fall times of 1 cycle was used. This means that the side lobes were below 60 dB, avoiding excitation of the cochlea beyond the characteristic frequency of the recording location. The length of the tone pips was long enough to allow the amplitudes of the 1st (0.25 kHz), 2nd (0.5 kHz), 3rd (1 kHz), 4th (2 kHz) and 5th harmonic (4 kHz) to be identified in the responses.

**Figure 2:**
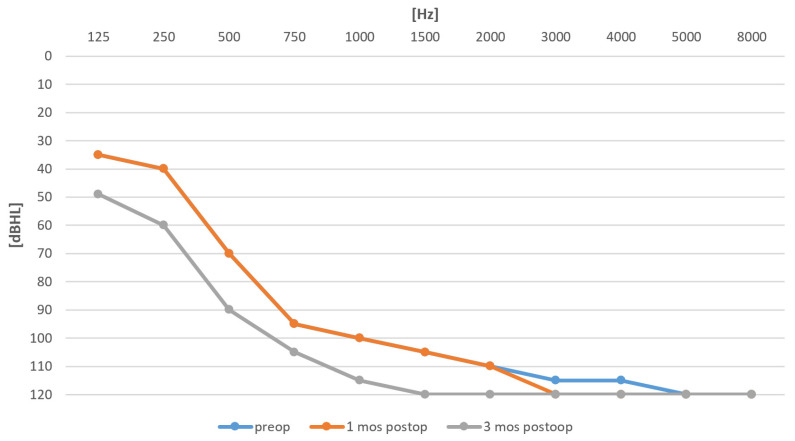
Preoperative audiogram (blue) and postoperative audiograms after 1 and 3 months (orange and grey).

Real-time measurements of each harmonic were made. The surgeon was instructed to gradually insert the electrode array at a speed of about 1.5 mm per 10 s. Continuous recordings in 13 ms windows were stored as sets of 100 (Figure 2). The real-time latency of the responses was measured when it reached 10% of the maximum amplitude as this is often measured as the latency of the response. Further details on the methodology and control measurements performed prior the study are given in Lorens et al. [3]. 

## 3. Results

Figure 2 compares the preoperative and postoperative audiograms. Some 3 weeks after the surgery, the postoperative audiogram was unchanged; however, after 3 months, a shift in pure tone average (PTA; 0.125–2 kHz) of 13 dB was observed.

For the preoperative CT, Otoplan v3.1 showed that the estimated insertion depth of the selected Flex 28 electrode array was 490°. This means that the tip of the array would reach the 500 Hz region in the cochlea. Table 1 is a record of the intraoperative events.

As shown in the table, continuous real-time monitoring starts at time 0 sec when the first two electrodes were inserted into the cochlea. Full insertion was achieved at 269 s. Then, sealing of the round window began, which lasted until 315 s. Recording finished at 440 s. 

**Figure 3:**
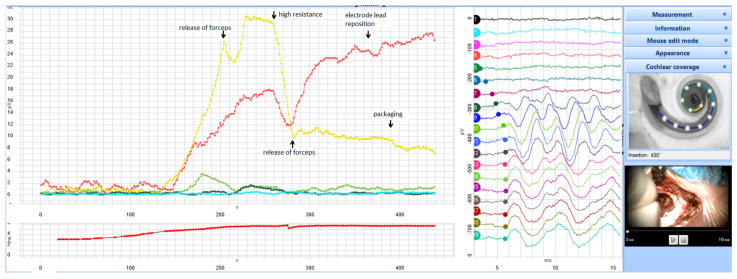
Real-time intraoperative ECochG monitoring (μV) during electrode insertion, along with highlighted surgical events from Table 1 (arrows). The main plot shows the amplitudes of each of the harmonics (red: 0.25 kHz; yellow: 0.5 kHz; light green: 1 kHz, dark green: 2 kHz, light blue: 4 kHz). The plot at bottom (red line) depicts the latency of the response. The numbered traces are continuous recordings from the most apical electrode. The top right image depicts the shows the estimated insertion angle of each of the 12 electrodes, up to 490° according to Otoplan v3.1.

Figure 3 displays the real-time intraoperative recordings. The most robust responses were measured for 0.5 kHz and 0.25 kHz. While the amplitude of the 0.5 kHz response was the highest from 220 to 250 s, the 250 Hz amplitude reached its highest response after the electrode was fully inserted and the sealing procedure had finished. The 1 kHz response was highest at 180 s. The 2 kHz response was highest at 230 s during the insertion. The 4 kHz response was not detectable at all during the surgery.

The postoperative CT revealed that the insertion depth was 465°. This was approximately 25° less than estimated prior to surgery. Table 2 sets out cochlear parameters. Table 3 includes the estimated preoperative insertion angles as well as postoperative measurements of the electrode contact angles. Interestingly, the insertion angles between the ipsilateral and contralateral side are very comparable (465 and 466°, respectively). 

**Figure 4:**
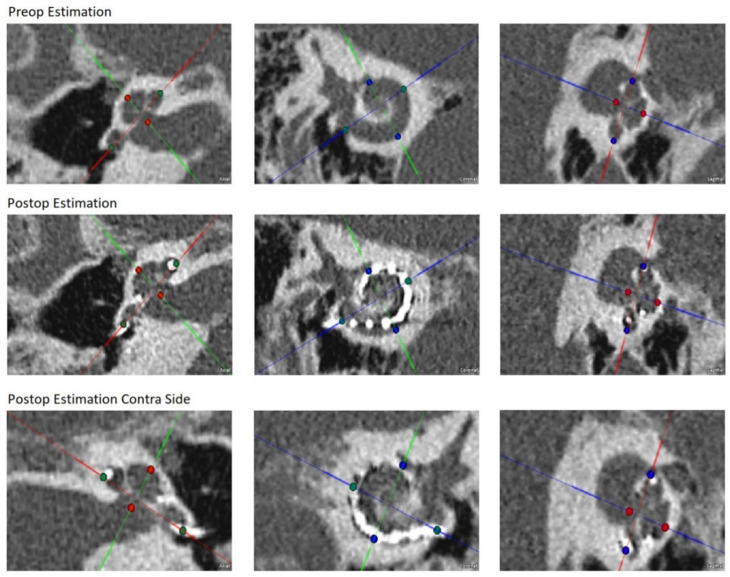
Preoperative and postoperative evaluation from Otoplan v.3.1.

The evaluation of the postoperative CT (Figure 4) suggests that electrode 4 was the closest to the 2 kHz characteristic frequency location, electrode 3 close to the 1 kHz, and electrode 2 close to the 0.5 kHz location. The 0.25 kHz characteristic frequency was not reached by any electrode. Based on this evaluation, the maximum amplitudes of the 1 kHz and 0.5 kHz traces seem to coincide with those of the characteristic frequencies.

Table 4 lists the maximum amplitudes of the 0.25, 0.5, 1, 2 and 4 kHz characteristic frequencies obtained during electrode insertion. The recording location of maximum amplitude for each specific characteristic frequency was estimated from the OR report and the surgical video. The electrode closest to each characteristic frequency was measured from the postoperative CT.

## 4. Discussion

Immediate postoperative hearing (3 weeks after surgery) of the subject was practically identical to preoperative hearing. Between then and 3 months postoperatively, a PTA (0.125–2 kHz) shift of 13.5 dB occurred. From our previous experience, such a shift is common during the first few months postoperatively [9,10,12]. 

The estimated preoperative insertion angles of each electrode were smaller than those measured from the postoperative CT. Such differences in the estimated insertion angles and postoperatively measured angles can occur if absolutely full electrode insertion is not reached. Whereas the Otoplan v 3.1 estimates electrode angles based on preoperative planning for full electrode insertion, the postoperative CT measurements are based on the actual insertion depth. In our case, the actual insertion depth was 27 mm instead of the absolute full insertion of 28 mm. This may suggest that the measured electrode angles are somewhat smaller than those estimated preoperatively (Table 2). Interestingly, this depth is very similar to the insertion depth in the contralateral ear (465° vs. 466°).

In the current case, we used a novel SPL Chirp signal for real-time intracochlear ECochG recordings during surgery, and in this way, we could monitor multiple frequency data in real time. We could identify the generation site of each ECochG signal, thus monitoring the integrity of multiple regions of the cochlea with different characteristic frequencies. From the evaluation of the postoperative CT, we were able to confirm that the maximum amplitudes of the 0.5 kHz and 1 kHz signals correlated with the appropriate regions in the cochlea. For the 0.25 kHz signal, its response was highest when the electrode was fully introduced into the cochlea, again confirming the tonotopicity of the cochlea since the furthermost electrode never reached the 0.25 kHz characteristic region. For the 2 kHz signal, it did not reach maximum amplitude at the expected 2 kHz characteristic region (instead, its maximum amplitude occurred at around 1 kHz characteristic frequency location) and this is probably because the response was very small, and we suspect this maximum may partially relate to 1 kHz auditory nerve neurophonic (ANN) potentials (Figure 3). A response at 4 kHz was not detected during the recordings. The relatively small amplitudes at both 2 and 4 kHz could be due to relatively poor cochlear health at these regions (for both these frequencies, the preoperative hearing thresholds were equal to or poorer than 110 dB HL). 

In Figure 1, the amplitudes of each of the harmonics of the ECochG signal (0.25, 0.5, 1, 2, and 4 kHz) are shown as a function of time of electrode insertion. The moments of closing of the round window and packing of the electrode lead are also marked. Based on the surgical video, we could precisely identify the surgical events that seemed to have an immediate effect on the health status of the cochlea (Table 1). By assuming that a drop in ECochG signal amplitude relates to EIT, and by noting the time of the particular surgical event associated with it, we can identify the following surgical events as a potential source of EIT (which are also shown as bold text in Table 1). These suspected surgical events were: 1) two particular cases of the electrode being released from the forceps; (2) insertion against high resistance; (3) electrode lead repositioning; (4) packing around the electrode lead. All can be detrimental to the health of the cochlea. One might infer that these types of EIT might be due to: (1) direct mechanical trauma from the electrode or from hydraulic forces; (2) compression or tearing of superficial arteries or veins; (3) damage to the lateral wall.

The amplitude of the 0.5 kHz response was highest from 220 to 250 s, dropped significantly while the electrodes were being inserted against high resistance, and did not recover after the final release of the forceps. In contrast, the 0.25 kHz signal reached its largest amplitude after the electrode was fully inserted and the sealing procedure was complete. There was a slight drop in amplitude during the insertion against high resistance, but there was subsequent full recovery and even a further gradual increase at the end of the recording (during packing around the electrode lead). 

The differences between the behavior of the 0.25 and 0.5 kHz signals can be explained by the position of the electrode in the cochlea: it was located in a region with a characteristic frequency of about 0.5 kHz and so was separated by some distance from the region with a characteristic frequency of 0.25 kHz. At the end of electrode insertion (between 245 and 268 s), a high resistance was reported. From the postop CT, it seems that the place of high resistance was in the region of cochlea with a characteristic frequency of 0.5 kHz. The insertion of the electrode against a resistance could introduce some trauma, as depicted in the drop of the ECochG response for 0.5 kHz as well as for 0.25 kHz. However, one can speculate that the nature of the trauma might be different in the region where the electrode was moving against a resistance (in the region tuned to 0.5 kHz) compared to the more apical region (with a characteristic frequency of 0.25 kHz), which the tip of the electrode did not reach. Therefore, after the drop in the amplitude of the 0.5 kHz response, it failed to recover, but in contrast, the amplitude of the 0.25 kHz response recovered and continued to grow.

Additionally, during the sealing of the round window, the micromovement of the electrode in the 0.5 kHz region could cause some trauma to this region of inner ear, which was reflected by a decrease in the 0.5 kHz ECochG response. The surgery video confirmed no electrode extrusion. During the sealing phase, there seemed to be no sign of trauma to the apical part of the cochlea, which the electrode did not reach, so the amplitude of the 0.25 kHz response could still grow. 

The presented case study can only be partially compared with previous studies, as in all other works, a single stimulation frequency has been used. However, the following paragraphs outline the main points of comparison. 

Campbell et al. [6] measured cochlear function using cochlear microphonics (CM) from the most apical electrode of a cochlear implant array during electrode insertion. Measurements were performed on 18 subjects using 0.5 kHz tone bursts. CMs were correlated with a surgery video and a postoperative audiogram. The authors found that in 47% of these implantations, there was transient or permanent reduction in CM amplitude. Patients with preserved CMs at the end of the insertion had, on average, 15 dB better low-frequency hearing preservation. The CM amplitude was most vulnerable during the last few millimeters of insertion or when inadvertent movement of the array occurred after full insertion.

Acharya et al. [13] recorded ECochG responses from two pediatric patients during electrode insertion as well as postoperatively. For both patients, good ECochG responses to 0.5 kHz stimuli were recorded at the end of the surgery and were later found to be unchanged postoperatively. Both patients had preserved hearing 3 months postoperatively. 

Dalbert et al. [7] performed promontory ECochG and intracochlear ECochG using the most apical electrode during cochlear implantation in 51 subjects. Intracochlear ECochG recordings during electrode insertion were obtained in three subjects using the most apical contact and a 0.5 kHz tone burst. For extracochlear recordings before and after electrode insertion, clicks and tone bursts of 0.25–1 kHz were used. In 11 subjects, a promontory stimulation (promstim) ECochG was recorded during electrode insertion. Changes of extracochlear ECochG recordings after full insertion of the CI electrode were correlated with pure-tone audiometric findings 4 weeks after surgery. The mean hearing loss in subjects who did not show a decrease in or loss of extracochlear ECochG signals during implantation was 12 dB, compared to a mean hearing loss of 22 dB in subjects where there was a detectable decrease in or a loss of ECochG signals. In extracochlear ECochG recordings, a mean increase in the ECochG signal of 4.4 dB occurred after opening the cochlea. O’Connell and colleagues [14] measured ECochG to 0.5 kHz tone bursts during electrode insertion in 18 subjects. The mean low-frequency pure tone average (LFPTA) increased following surgery by an average of 28 dB (range of 8–50 dB). The threshold elevation was significantly greater for electrodes that involved scalar dislocation. No correlation was found between intraoperative ECochG and postoperative behavioral thresholds collapsed across frequency; however, the mean differences in thresholds measured by intraoperative ECochG and postoperative audiometry were significantly smaller when electrodes were inserted completely within scala tympani (ST) vs. those translocating from ST to scala vestibuli. A significant correlation was observed between postoperative ECochG thresholds and behavioral thresholds obtained at activation.

Haumann at al. [15] recorded intraoperative ECochGs using extracochlear and intracochlear electrodes in 10 adults. Prior to electrode insertion, extracochlear recordings to low-frequency tone bursts were measured. During insertion, extracochlear recordings to 0.5 and 1 kHz stimuli were recorded. Following electrode insertion, intracochlear and extracochlear ECochG responses to low-frequency tonebursts at the most apical electrode were performed. The authors concluded that intraoperative intracochlear ECochG thresholds were highly correlated to preoperative audiometric thresholds at 0.25 and 0.5 kHz. However, the correlations of both intraoperative ECochG recordings to postoperative pure tone thresholds were low.

Harris et al. [16] performed continuous intraoperative intracochlear ECochG recording during electrode insertion and packing on 14 subjects. The stimuli were tone bursts of 0.5 kHz with the continuous evaluation of CM and ANN during electrode insertion. The 1st harmonic amplitude generally increased steadily with electrode insertion. 

Finally, the application of continuous intraoperative monitoring by the use of multifrequency tone bursts was proposed by Barnes et al. [17]; however no comparative results were shown.

## 5. Conclusions

Real-time intraoperative monitoring, with the use of multiple characteristic frequencies and video recording, may allow surgeons and audiologists to continuously assess cochlear function. With the right tools, experience with the technique is not required. Such an ECochG monitoring tool may prove useful during cochlear implantation surgery to allow the frequency-specific monitoring of the patient’s hearing status for minimizing electrode insertion trauma. This may be critical for improved hearing outcomes after cochlear implantation.

## Figures and Tables

**Table 1:** Intraoperative surgical events over 440 s. For better visualization time scale was highlighted.

Time [s]	0	14	25	34	50	49–59	68	75–78
**Surgical Event**	2 electrodes introduced	Electrode slips out of forceps	3 electrodes introduced	Slight change of direction and change of side of forceps	4 electrodes introduced	Small resistance during insertion	Slight change of direction and change of side of forceps	Small resistance during insertion
**Time [s]**	75	85	90–91	103	103 until the end	123	123–140	141–145
**Surgical Event**	6 electrodes introduced	Slight change of direction and change of side of forceps	Electrode released from the hand	7 electrodes introduced	Electrode released from the hand	8 electrodes introduced	Electrode released from forceps	Small resistance during insertion
**Time [s]**	145	148–163	170–171	173	173–192	198–203	203	203–238
**Surgical Event**	9 electrodes introduced	Electrode released from forceps	Small resistance during insertion	10 electrodes introduced	**Electrode released from forceps**	Moderate resistance during insertion	11 electrodes introduced	Electrode released from forceps
**Time [s]**	245–268	269	269	289–298	311–315	333–338	338–433	440
**Surgical Event**	**High resistance during insertion**	**Moment of releasing forceps**	Full electrode insertion	Sealing of round window with the first periosteum fragment	Sealing of round window with the second periosteum fragment	**Electrode lead repositioning**	**Packing around electrode lead**	Recording finished

**Table 2:** Cochlear parameters.

	Diameter (A) (mm)	Height (H) (mm)	Width (B) (mm)	Estimated CDL (mm)	Estimated CDL from RW (mm)
Preop estimate	9.3	4.0	7.5	38.7	36.2
Postop measurement	9.8	4.1	7.6	39.6	37.1
Postop measurement contra side	9.9	4.1	7.6	39.7	37.2

**Table 3:** Preoperative insertion angle estimates and postoperative measurements of electrode contact angles. The angle is measured from the middle of the RW.

Electrode	1	2	3	4	5	6	7	8	9	10	11	12
Preop angle estimate [°]	488	430	375	327	284	243	204	166	131	98	67	42
Postop angle measurement [°]	465	403	345	295	253	219	185	151	113	75	36	11
Postop angle measurement contra side [°]	466	403	346	297	258	228	197	163	129	92	55	21

**Table 4:** Maximum amplitudes of characteristic frequencies 0.25, 0.5, 1, 2, and 4 kHz obtained during electrode insertion. Recording locations are rounded to the nearest millimeter. Electrode 1 is the most apical electrode.

Frequency (kHz)	0.25	0.5	1	2	4
Time (s)	440	220–250	180	230	Not detected
Maximum amplitude (mm)	full insertion	11 electrodes inserted	10 electrodes inserted	11 electrodes inserted	Not detected
Location of maximum amplitude (mm)	26	22	20	18	11
Closest electrode to characteristic frequency	Electrode 1	Electrode 2	Electrode 3	Electrode 4	Electrode 7

## Data Availability

Not applicable.
